# Effects of Low-Dose Spironolactone Combined with Metformin or Either Drug Alone on Insulin Resistance in Patients with Polycystic Ovary Syndrome: A Pilot Study

**DOI:** 10.1155/2022/9927240

**Published:** 2022-03-19

**Authors:** Tao Long, Ying Zhang, Chunping Zeng, Siyuan Zheng, Lin Zhou, Haiyan Liu

**Affiliations:** ^1^Endocrinology Department, The Fifth Affiliated Hospital of Guangzhou Medical University, Guangzhou, China; ^2^Endocrinology Department, The Third Affiliated Hospital of Guangzhou Medical University, Guangzhou, China

## Abstract

Metformin and spironolactone alone can be used for the management of polycystic ovarian syndrome (PCOS), and their combination could result in even better outcomes. To compare the effects and safety of low-dose spironolactone combined with metformin or either drug alone on insulin resistance (IR) and functional improvement in patients with PCOS, this was a single-center, randomized, open-label, pilot study of patients with PCOS at the Third Affiliated Hospital of Guangzhou Medical University between 01/2014 and 01/2016. The participants were randomized 1 : 1 : 1 to metformin, spironolactone, or metformin + spironolactone. The primary endpoint was the change in the homeostatic model assessment (HOMA)-IR after 12 weeks of treatment. A total of 189 participants were randomized (63 per group); 31 dropped out, and 54, 51, and 53 completed the 12-week treatment in the metformin, spironolactone, and combined groups, respectively. There were no differences in any parameters between the metformin and spironolactone groups (all *P* > 0.05). In the combined group, after 12 weeks of treatment, HOMA-IR (1.71 ± 0.91) was lower than in the metformin (1.92 ± 1.07, *P* < 0.05) and spironolactone (2.38 ± 1.14, *P* < 0.05) groups. In addition, total testosterone (TT), free androgen index (FAI), and area under the curve-insulin (AUCins) were lower in the combined group compared with the metformin group (all *P* < 0.05), while TT, FAI, HOMA-*β*, fasting plasma glucose, and AUCins were lower in the spironolactone group (all *P* < 0.05). Both metformin and spironolactone decreased HOMA-IR in patients with PCOS but without differences between the two monotherapies. The combined therapy decreased HOMA-IR to a greater extent than monotherapy.

## 1. Introduction

Polycystic ovary syndrome (PCOS) consists of the clinical findings of ovulatory dysfunction, hyperandrogenism, and polycystic ovaries [1–3]. It is often associated with complications, including obesity, hyperinsulinemia, and infertility and is the most common endocrine disorder in women of reproductive age [2–4]. The exact criteria for the diagnosis are variable, leading to rates of 5%–21% [5]. Up to 28% of overweight women have PCOS [6]. In women with PCOS, the normal interplay of the sexual hormones controlling ovulation is dysregulated [3]. In addition, peripheral insulin resistance (IR) is observed in most women with PCOS, either lean or overweight [7, 8], and will contribute to excess androgens, which will disrupt fertility [3, 9]. The risk factors for PCOS include a family history of PCOS [10], environmental toxins, and advanced glycation end-products [11].

In addition to the use of exogenous sex hormone drugs to regulate menstruation and improve hirsutism in women with PCOS, metformin, a classic drug for the treatment of diabetes, has been used as first-line therapy for PCOS [12, 13]. Indeed, metformin has an insulin-sensitizing effect and will lead to IR relief, weight loss, improved oligomenorrhea, and alleviated hirsutism [14]. In addition to its diuretic effect, spironolactone is considered to have antiandrogenic effects and improve hirsutism and acne [15, 16].

Currently, there are few prospective studies on metformin combined with low-dose spironolactone in the treatment of PCOS. Numerous previous studies have fully demonstrated the improvement effects of metformin on IR, obesity, and inflammation in patients with PCOS [12, 13], as well as the effects of spironolactone [15, 16]. Both drugs alone are effective in the management of PCOS, and spironolactone could be better for the management of hirsutism, menstrual cycle issues, and hormonal perturbations [17], but the combination of the two drugs could result in even better outcomes in women with PCOS [16].

Therefore, this study was designed to compare the effects of low-dose spironolactone combined with metformin or either drug alone on IR and functional improvement in patients with PCOS, as well as compliance, safety, and incidence of adverse effects through a prospective randomized open-label study. The results should provide additional rationale for the use of those drugs in patients with PCOS.

## 2. Materials and Methods

### 2.1. Study Design and Participants

This was a single-center, randomized, open-label, pilot study. The participants were patients with PCOS admitted to the outpatient and inpatient departments of the Third Affiliated Hospital of Guangzhou Medical University between January 2014 and January 2016. This study was approved by the Ethics Committee of the Third Affiliated Hospital of Guangzhou Medical University (approval number 2018061). Written informed consent was obtained from all participants.

PCOS was diagnosed according to the Rotterdam Diagnostic Criteria for PCOS developed by the European Society for Human Reproduction and Embryology (ESHRE) and the American Society of Reproductive Medicine (ASRH) in 2003 [18]: (1) oligo- or anovulation; (2) clinical and/or biochemical signs of hyperandrogenism such as hirsutism, acne, androgenetic alopecia, and elevated levels of serum total testosterone or free testosterone; (3) polycystic ovaries, namely unilateral ovarian volume enlargement by more than 10 ml (excluding cysts and dominant follicles) or 12 or more follicles of 2–9 mm in diameter in the unilateral ovary; (4) meeting two of the above three criteria after the exclusion of diseases that cause elevated androgen levels, such as congenital adrenal hyperplasia, Cushing's syndrome, androgen-secreting tumors, and those that led to ovulation disorders, such as hyperprolactinemia, pituitary, or hypothalamic amenorrhea, thyroid dysfunction, and primary ovarian insufficiency.

The inclusion criteria were (1) age >18 years; (2) history of sexual life; and (3) agreed to use barrier contraception within 12 weeks. The exclusion criteria were (1) other endocrine diseases such as hyperprolactinemia and congenital adrenal hyperplasia (CAH), or hyperprolactinemia and other endocrine diseases that led to hyperandrogenism, such as Cushing's syndrome, CAH, and androgen-secreting tumors; (2) patients with immune diseases, cancer, type 1 diabetes, or history of type 2 diabetes; (3) medications within 12 weeks, including cortisol, antidepressants, hypoglycemic agents, hormonal contraceptives, ovulation-inducing drugs, or other drugs that affect the metabolism of glycolipids and sex hormones; (4) pregnant or lactating women within the recent 6 months or those with pregnancy plan within 3 months; (5) patients with speech impairment or those with disabilities who cannot understand the experimental requirements; (6) patients with severe organ failure such as liver or renal function, or mental disorders; (7) patients with immunodeficiency or HIV infection; (8) history of drug abuse and alcohol dependence in the past 5 years; (9) history of pancreatitis or pancreatectomy; or 10) participated in any clinical trials within 3 months.

### 2.2. Randomization

The participants were randomized 1 : 1 : 1 to one of three groups according to a random number table: the metformin group, spironolactone group, and metformin + spironolactone group. This was an open-label study.

### 2.3. Treatment

The subjects were given the life style guidance of the unified scheme. Guidance includes diet control and simple exercise prescription under the guidance of a nutritionist and smoking cessation and alcohol prohibition.

The metformin group was treated with metformin 1500 mg/d (Bristol-Meyer Squibb, New York, NY, USA). The spironolactone group was given spironolactone 40 mg/d (Hangzhou Minsheng Pharmaceutical Co., Ltd., China). The combined therapy group was treated with metformin 1500 mg/d and spironolactone 40 mg/d. The treatment period was 12 weeks.

### 2.4. Endpoints

The primary endpoint was the change in the homeostatic model assessment (HOMA)-IR after 12 weeks of treatment. The secondary endpoints included the changes in the other glucose homeostasis assessments, compliance, safety, changes in body mass index (BMI), and changes in the free androgen index (FAI).

### 2.5. Data Collection

The participants' anthropometry and laboratory indexes were recorded at baseline and the end of the treatment. Anthropometry included height, weight, waist and hip circumference, and blood pressure. The modified Ferriman–Gallwey score (mF-G score), Rosenfield score, menstrual status, and the number of menstruation in the recent 12 months were recorded.

All participants underwent an oral anhydrous glucose tolerance test (OGTT) of 75 g. The participants started fasting at 22 : 00 the night before the test. They were allowed to drink water, but any calorie intake was forbidden. After fasting for at least 10 h, OGTT was performed at approximately 7 : 00 the next morning, using 75 g of anhydrous glucose dissolved in 300 ml of water, which was drunk within 3–5 min. Other foods, medicines, or smoking were forbidden. Sitting still or walking and having rest were recommended, and strenuous exercise and other strong stimulations were avoided. At 0, 0.5, 1, 2, and 3 hours after taking glucose solution (recording from the first oral intake of the glucose solution), venous blood was collected for measurement of blood glucose (fasting plasma glucose (FPG), 0.5 h postprandial glucose (PPG), 1 h PPG, 2 h PPG, 3 h PPG) and insulin (INS) levels.

The venous blood of the subject was used to measure luteinizing hormone (LH), follicle-stimulating hormone (FSH), total cholesterol (TC), sex hormone-binding globulin (SHBG), and total testosterone (TT).

### 2.6. Definitions

BMI was calculated as weight (kg)/height (m^2^). The waist-hip ratio (WHR) was calculated as the waist circumference (cm)/hip circumference (cm). HOMA-IR was calculated as fasting INS (FINS) (mU/L) × FPG (mmol/L)/22.5. HOMA-*β* was calculated as 20 × FINS (mIU/L)/(FPG (mmol/L) − 3.5). The quantitative INS sensitivity check index (QUICKI) for the assessment of insulin sensitivity was calculated as 1/(logFPG + logFINS). Adult QUICKI <0.357 was the diagnostic criterion of IR [19]. The OGTT area under curve-glucose (AUCglu) was calculated as (FPG + 3 hPPG)/2 + 1 hPPG + 2 hPPG. (glucose unit: mmol/L). The OGTT area under curve-insulin (AUCins) was calculated as (FINS + 3 hINS)/2 + 1 hINS + 2 hINS. (glucose unit: mmol/L, insulin unit: mIU/L). The FAI was calculated as [(TT (nmol/L) × 100)/SHBG (nmol/L)].

### 2.7. Safety

All subjects were followed at the outpatient clinic at 4, 8, and 12 weeks after starting the study medication to record patients' compliance and adverse events. During the follow-up, we monitored relevant safety indicators, including gastrointestinal reactions, electrolyte disorders, liver and kidney dysfunction, and any other clinically significant discomfort.

### 2.8. Statistical Analysis

Statistical analyses were performed using SPSS 19.0 for Windows (IBM, Armonk, NY, USA). The Shapiro–Wilk method was used to test whether the data were normally distributed. Continuous data with a normal distribution were presented as means ± standard deviations, and those not conforming to normal distribution were log-transformed. The continuous data within groups and among the three groups at baseline and 12 weeks after medication were compared by one-way analysis of variance (ANOVA) or the Kruskal–Wallis method. The changes in indicators at baseline and 12 weeks after medication in the same treatment group were analyzed by single-factor repeated measures ANOVA with the LSD post hoc test. *P* < 0.05 was considered statistically significant.

## 3. Results

### 3.1. Characteristics of the Participants

**Figure 1:**
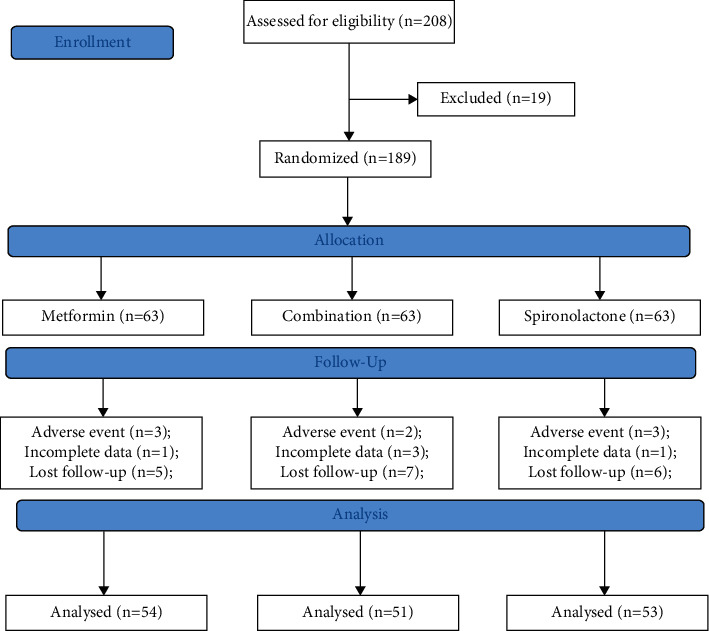
Participant flowchart.

Figure 1 presents the participant flowchart. A total of 208 participants were eligible, but 19 declined participation; 189 were randomized (63/group). A total of 31 patients dropped out, and 54, 51, and 53 completed the 12-week treatment in the metformin, spironolactone, and combined groups, respectively.  Table 1 presents the characteristics of the participants.

### 3.2. Effect of the Treatments

In the metformin group, after 12 weeks of treatment, weight was decreased (from 63.2 ± 12.4 to 61.8 ± 11.6 kg, *P* < 0.05), LH/FSH was decreased (from 1.67 ± 0.95 to 1.40 ± 0.90, *P* < 0.05), TC was decreased (from 4.81 ± 1.01 to 4.46 ± 0.68 mmol/L, *P* < 0.05), TT was decreased (from 2.25 ± 1.25 to 2.05 ± 0.89 nmol/L, *P* < 0.05), FAI was decreased (from 6.71 ± 8.87 to 5.78 ± 7.62, *P* < 0.05), QUICKI was increased (from 0.33 ± 0.03 to 0.36 ± 0.03, *P* < 0.05), HOMA-IR was decreased (from 3.30 ± 2.34 to 1.92 ± 1.07, *P* < 0.05), FINS was decreased (from 14.95 ± 9.61 to 9.14 ± 4.81 mU/L, *P* < 0.05), AUCins was decreased (from 289.6 ± 151.0 to 273.3 ± 131.0, *P* < 0.05), and AUGglu was decreased (from 22.8 ± 5.4 to 20.6 ± 4.3, *P* < 0.05) (Table 2).

In the spironolactone group, after 12 weeks of treatment, weight was decreased (from 65.5 ± 16.8 to 63.7 ± 16.2 kg, *P* < 0.05), LH/FSH was decreased (from 1.86 ± 0.90 to 1.59 ± 0.81, *P* < 0.05), TT was decreased (from 2.09 ± 0.80 to 1.79 ± 0.69 nmol/L, *P* < 0.05), FAI was decreased (from 6.99 ± 5.26 to 4.88 ± 4.20, *P* < 0.05), HOMA-IR was decreased (from 3.02 ± 1.90 to 2.38 ± 1.14, *P* < 0.05), FINS was decreased (from 13.87 ± 8.08 to 10.62 ± 4.77 mU/L, *P* < 0.05), and AUCins was decreased (from 271.2 ± 146.4 to 271.2 ± 143.2, *P* < 0.05) (Table 2).

In the combined group, after 12 weeks of treatment, LH/FSH was decreased (from 2.06 ± 0.99 to 1.92 ± 0.99, *P* < 0.05), TT was decreased (from 2.50 ± 1.03 to 1.88 ± 0.60 nmol/L, *P* < 0.05), FAI was decreased (from 7.07 ± 4.21 to 3.58 ± 3.0, *P* < 0.05), QUICKI was increased (from 0.34 ± 0.03 to 0.36 ± 0.03, *P* < 0.05), HOMA-IR was decreased (from 2.47 ± 1.63 to 1.71 ± 0.91, *P* < 0.05), HOMA-*β* was increased (from 179.9 ± 99.0 to 973.7 ± 12645, *P* < 0.05), FINS was decreased (from 11.24 ± 7.10 to 8.15 ± 3.92 mU/L, *P* < 0.05), AUCins was decreased (from 267.8 ± 126.0 to 232.1 ± 121.3, *P* < 0.05), and AUGglu was decreased (from 20.5 ± 5.0 to 17.5 ± 4.2, *P* < 0.05) (Table 2).

Two by two differences in variations comparison among the three groups: compared with SPI group and met group, the levels of HOMA-IR, TT, FAI, and AUCIns decreased more significantly in com group. Compared with the SPI group, two by two comparison among the three groups: compared with SPI group and met group, the levels of HOMA-IR, TT, FAI, and AUCIns decreased more significantly in com group. Compared with the SPI group, the level of B increased more significantly in the com group (*P* < 0.05).

Table 3 shows that there were no differences in any parameters between the metformin and spironolactone groups (all *P* > 0.05). In the combined group, after 12 weeks of treatment, TT, FAI, HOMA-IR, and AUCins were all lower than in the metformin group (all *P* < 0.05), while TT, FAI, HOMA-IR, HOMA-*β*, FPG, and AUCins were lower than in the spironolactone group (all *P* < 0.05).

### 3.3. Safety

We performed liver and kidney function and electrolyte tests at baseline and during follow-up at 4 and 12 weeks after enrollment. No abnormality was found in all subjects. As seen in Table 4, there were six participants with nausea, one with vomiting, and three with diarrhea in the metformin group. In the spironolactone group, there were one participant with polyuria, three with nausea, one with diarrhea, and two with dry mouth. In the combined group, there were five participants with nausea, one with vomiting, and three with diarrhea. Most participants in the three groups tolerated the medication after about 2–4 weeks. Those who were unable to tolerate the side effects withdrew from the study, including three in the metformin group, two in the spironolactone group, and two in the combined group. The dose of spironolactone in this study was 40 mg/d. None of the patients had serious adverse events, such as hyperkalemia or elevated levels of creatinine or urea nitrogen. Compared with the monotherapy groups, the incidence of adverse events was not higher in the combined group.

## 4. Discussion

Metformin and spironolactone alone can be used for the management of polycystic ovarian syndrome [12, 13, 15, 16], and their combination could result in even better outcomes [16]. Therefore, this study aimed to compare the effects and safety of low-dose spironolactone combined with metformin or either drug alone on IR and functional improvement in patients with PCOS. The results showed that both metformin and spironolactone decreased HOMA-IR in patients with PCOS but without differences between the two monotherapies. The combined therapy decreased HOMA-IR to a greater extent than monotherapy. The bodyweight of subjects in the com group was lower than that of the other two groups, although BMI and HOMA-IR were similar at baseline, and there was no statistically significant difference. However, relatively lower body weight may affect the conclusion of HOMA-IR changes. Clinically, lower body weight sometimes represents lower IR. Com group achieved greater HOMA-IR changes than the other two groups on the basis of lower body weight. We believe that this result still has certain clinical significance.

Two studies by Ganie et al. showed that both metformin and spironolactone were effective for the management of PCOS [16, 17], but that spironolactone had better effects on hirsutism, menstrual cycle issues, and hormonal perturbations. Alpañés et al. showed that spironolactone combined with an oral contraceptive led to greater decreases in androgens than metformin [20], but without differences in glucose intolerance. Kulshreshtha et al. showed that there were no differences in OGTT parameters between metformin and spironolactone [21]. In the present study, both monotherapies improved glucose metabolism and decreased TT, but no differences were observed in any of the variables, including parameters of glucose metabolism, as well as factors associated with PCOS, such as the number of menstrual cycles per year and hirsutism. Spironolactone may lead to adverse reactions such as irregular menstruation and abnormal uterine bleeding during the intermenstrual period, but given irregular menstruation was present in most eligible patients at baseline, and the observation period was short (12 weeks vs. 6 months in Ganie et al.) [17], it could be considered that the possibility of drug-induced irregular menstruation was small. Thus, no evaluation was performed in this study.

On the other hand, the present study showed that the combination therapy had better outcomes than monotherapy in glucose metabolism parameters and androgens (FF and FAI). This is supported by a study by Ganie et al. in 204 women [16]. On the other hand, Diri et al. showed that the combination of metformin and spironolactone was not better than spironolactone alone in terms of hormone levels and insulin resistance [22]; this previous study included a small number of participants (<20/group), but their treatment lasted 12 months.

Those effects are globally supported by the known effects of the drugs. Indeed, metformin is a well-known insulin sensitizer that also decreases androgen production [17, 23–25]. Spironolactone is also known for its antiandrogen effects [17, 26], and it is also able to improve metabolic parameters such as glucose tolerance and cholesterol levels [27, 28]. The aim of combining an insulin sensitizer to an antiandrogen is to simultaneously improve multiple parameters observed in PCOS. Previous studies examined various combinations of such drugs [29–35].

In the present study, the hirsutism scores were not changed in any group, while previous studies showed that spironolactone, metformin, and their combination improved hirsutism [16, 17, 22, 36]. This could be due to the relatively short treatment course in the present study compared with those previous studies and the fact that the women included here had relatively low hirsutism at baseline.

In the present study, compliance and safety were similar across the three groups. Some patients discontinued treatments because of inconvenient and uncomfortable adverse reactions, but none had serious adverse events. Such a good safety profile of the combination is similar to that observed in previous studies [16, 17, 22, 36].

The present study has limitations. No sample size was originally calculated, and a convenient sample size of 200 patients was determined for screening. In addition, this was a single-center trial, and the follow-up was short. When we included the patients, we did not specify the level of insulin resistance in the patient. Additional studies might be necessary for determining the benefits of the metformin-spironolactone combination in PCOS.

## 5. Conclusions

In conclusion, both metformin and spironolactone decreased HOMA-IR in patients with PCOS but without differences between the two monotherapies. The combined therapy decreased HOMA-IR to a greater extent than monotherapy.

## Figures and Tables

**Table 1:** Baseline characteristics of the participants.

	MET group (*n* = 54)	SPI group (*n* = 53)	COM (*n* = 51)	*P*
Age (years)	27.0 ± 3.7	27.6 ± 3.7	27.2 ± 3.6	>0.05
Number of menstruation per year (normal ≥8/year)	5.7 ± 2.1	6.1 ± 1.9	5.8 ± 1.8	>0.05
SBP (mm Hg)	112 ± 11	124 ± 13	117 ± 9	>0.05
DBP (mm Hg)	72 ± 13	79 ± 10	76 ± 8	>0.05
Weight (kg)	63.2 ± 12.4	65.5 ± 16.8	56.5 ± 9.0	<0.05
BMI (kg/m^2^)	25.6 ± 4.5	25.9 ± 6.7	25.4 ± 3.7	>0.05
WHR	0.87 ± 0.07	0.87 ± 0.09	0.86 ± 0.09	>0.05
LH/FSH	1.67 ± 0.95	1.86 ± 0.90	2.06 ± 0.99	>0.05
TC (mmol/L)	4.81 ± 1.01	4.91 ± 1.40	4.74 ± 0.98	>0.05
TT (nmol/L)	2.25 ± 1.25	2.09 ± 0.80	2.50 ± 1.03	>0.05
FAI	6.71 ± 8.87	6.99 ± 5.26	7.07 ± 4.21	>0.05
QUICKI	0.33 ± 0.03	0.34 ± 0.03	0.34 ± 0.03	>0.05
HOMA-IR	3.30 ± 2.34	3.02 ± 1.90	2.47 ± 1.63	>0.05
HOMA-*β*	224.6 ± 166.8	299.6 ± 381	179.9 ± 99	>0.05
FPG (mmol/L)	4.94 ± 0.63	4.79 ± 0.55	4.84 ± 0.61	>0.05
FINS (mU/L)	14.95 ± 9.61	13.87 ± 8.08	11.24 ± 7.1	>0.05
mF-G score	4.2 ± 2.1	4.2 ± 1.3	4.1 ± 1.4	>0.05
AUCins	289.6 ± 151	282.5 ± 146.4	267.8 ± 126	>0.05
AUCglu	22.8 ± 5.4	21.0 ± 4.8	20.5 ± 5.0	>0.05
Rosenfield score	0.89 ± 1.01	0.90 ± 0.92	0.85 ± 1.05	>0.05

SBP: systolic blood pressure; DBP: diastolic blood pressure; BMI: body mass index; WHR: waist-hip ratio; LH/FSH: luteinizing hormone/follicular-stimulating hormone; TC: total cholesterol; TT: total testosterone; FAI: free androgen index; QUICKI: quantitative insulin sensitivity check index; HOMA: homeostatic model assessment; IR: insulin resistance; FPG: fasting plasma glucose; FINS: fasting insulin; mG-G score: modified Ferriman–Gallwey score; AUCglu: oral glucose tolerance test area under curve-glucose; AUCins: oral glucose tolerance test area under curve-insulin.

**Table 2:** Comparison of the three groups before and after treatment.

	MET group (*n* = 54)		SPI group (*n* = 53)		COM group (*n* = 51)	
	Week 0	Week 12	Week 0	Week 12	Week 0	Week 12
Number of menstruation per year (normal ≥8/year)	5.7 ± 2.1	5.9 ± 2.1	6.1 ± 1.9	6.9 ± 2.4	5.8 ± 1.8	6.1 ± 2.3
SBP (mm Hg)	112 ± 11	112 ± 11	124 ± 13	123 ± 11	117 ± 9	117 ± 8
DBP (mm Hg)	72 ± 13	72 ± 12	79 ± 10	78 ± 8	76 ± 8	74 ± 7
Weight (kg)	63.2 ± 12.4	61.8 ± 11.6^a^	65.5 ± 16.8	63.7 ± 16.2^a^	56.5 ± 9.0	54.6 ± 8.8
BMI (kg/m^2^)	25.6 ± 4.5	25.0 ± 4.2	25.9 ± 6.7	25.2 ± 6.5	25.4 ± 3.7	24.7 ± 3.6
WHR	0.87 ± 0.07	0.83 ± 0.04	0.87 ± 0.09	0.82 ± 0.07	0.86 ± 0.09	0.81 ± 0.10
LH/FSH	1.67 ± .95	1.4 ± 0.90^a^	1.86 ± 0.90	1.59 ± 0.81^a^	2.06 ± 0.99	1.92 ± 0.99^a^
TC (mmol/L)	4.81 ± 1.01	4.46 ± 0.68^a^	4.91 ± 1.40	4.75 ± 0.98	4.74 ± 0.98	4.64 ± 0.71
TT (nmol/L)	2.25 ± 1.25	2.05 ± 0.89^a^	2.09 ± 0.80	1.79 ± 0.69^a^	2.50 ± 1.03	1.88 ± 0.60^a^
FAI	6.71 ± 8.87	5.78 ± 7.62^a^	6.99 ± 5.26	4.88 ± 4.2^a^	7.07 ± 4.21	3.58 ± 3.0^a^
QUICKI	0.33 ± 0.03	0.36 ± 0.03^a^	0.34 ± 0.03	0.34 ± 0.02	0.34 ± 0.03	0.36 ± 0.03^a^
HOMA-IR	3.30 ± 2.34	1.92 ± 1.07^a^	3.02 ± 1.90	2.38 ± 1.14^a^	2.47 ± 1.63	1.71 ± 0.91^a^
HOMA-*β*	224.6 ± 166.8	1978.0 ± 7068	299.6 ± 381	867.76 ± 651	179.9 ± 99	973.7 ± 1264^a^
FBG (mmol/L)	4.94 ± 0.63	4.65 ± 0.45	4.79 ± 0.55	5.02 ± 0.58	4.84 ± 0.61	4.68 ± 0.58
FINS (mU/L)	14.95 ± 9.61	9.14 ± 4.81^a^	13.87 ± 8.08	10.62 ± 4.77^a^	11.24 ± 7.1	8.15 ± 3.92^a^
mF-G score	4.2 ± 2.1	3.9 ± 1.3	4.2 ± 1.3	4.1 ± 1.5	4.1 ± 1.4	4.0 ± 1.2
AUCins	289.6 ± 151	273.3 ± 131^a^	282.5 ± 146.4	271.2 ± 143.2^a^	267.8 ± 126	232.1 ± 121.3^a^
AUCglu	22.8 ± 5.4	20.6 ± 4.3^a^	21.0 ± 4.8	19.2 ± 4.6	20.5 ± 5.0	17.5 ± 4.2^a^
Rosenfield score	0.89 ± 1.01	0.84 ± 0.98	0.90 ± 0.92	0.88 ± 0.90	0.85 ± 1.05	0.83 ± 1.00

^a^ *P* < 0.05 week 0 vs. week 12. MET: metformin; SPI: spironolactone; COM: combined; SBP: systolic blood pressure; DBP: diastolic blood pressure; BMI: body mass index; WHR: waist-hip ratio; LH/FSH: luteinizing hormone/follicular-stimulating hormone; TC: total cholesterol; TT: total testosterone; FAI: free androgen index; QUICKI: quantitative insulin sensitivity check index; HOMA: homeostatic model assessment; IR: insulin resistance; FPG: fasting plasma glucose; FINS: fasting insulin; mG-G score: modified Ferriman–Gallwey score; AUCglu: oral glucose tolerance test area under curve-glucose; AUCins: oral glucose tolerance test area under curve-insulin.

**Table 3:** Comparison of the outcomes among the three groups after 12 weeks of treatment.

	MET group (*n* = 54)	SPI group (*n* = 53)	COM group (*n* = 51)
Number of menstruation per year (normal ≥8/year)	5.9 ± 2.1	6.9 ± 2.4	6.1 ± 2.3
SBP (mm Hg)	112 ± 11	123 ± 11	117 ± 8
DBP (mm Hg)	72 ± 12	78 ± 8	74 ± 7
Weight (kg)	61.8 ± 11.6	63.7 ± 16.2	54.6 ± 8.8
BMI (kg/m^2^)	25.0 ± 4.2	25.2 ± 6.5	24.7 ± 3.6
WHR	0.83 ± 0.04	0.82 ± 0.07	0.81 ± 0.10
LH/FSH	1.40 ± 0.90	1.59 ± 0.81	1.92 ± 0.99
TC (mmol/L)	4.46 ± 0.68	4.75 ± 0.98	4.64 ± 0.71
TT (nmol/L)	2.05 ± 0.89	1.79 ± 0.69	1.88 ± 0.60^ab^
FAI	5.78 ± 7.62	4.88 ± 4.2	3.58 ± 3.0^ab^
QUICKI	0.36 ± 0.03	0.34 ± 0.02	0.36 ± 0.03
HOMA-IR	1.92 ± 1.07	2.38 ± 1.14	1.71 ± 0.91^ab^
HOMA-*β*	1978.0 ± 7068	867.76 ± 651	973.7 ± 1264^b^
FBG (mmol/L)	4.65 ± 0.45	5.02 ± 0.58	4.68 ± 0.58^b^
FINS (mU/L)	9.14 ± 4.81	10.62 ± 4.77	8.15 ± 3.92
mF-G score	3.9 ± 1.3	4.1 ± 1.5	4.0 ± 1.2
AUCins	273.3 ± 131	271.2 ± 143.2	232.1 ± 121.3^ab^
AUCglu	20.6 ± 4.3	19.2 ± 4.6	17.5 ± 4.2
Rosenfield score	0.84 ± 0.98	0.88 ± 0.90	0.83 ± 1.00

^a^ *P* < 0.05 vs. metformin. ^b^*P* < 0.05 vs. spironolactone. MET: metformin; SPI: spironolactone; COM: combined; SBP: systolic blood pressure; DBP: diastolic blood pressure; BMI: body mass index; WHR: waist-hip ratio; LH/FSH: luteinizing hormone/follicular-stimulating hormone; TC: total cholesterol; TT: total testosterone; FAI: free androgen index; QUICKI: quantitative insulin sensitivity check index; HOMA: homeostatic model assessment; IR: insulin resistance; FPG: fasting plasma glucose; FINS: fasting insulin; mG-G score: modified Ferriman–Gallwey score; AUCglu: oral glucose tolerance test area under curve-glucose; AUCins: oral glucose tolerance test area under curve-insulin.

**Table 4:** Compliance among the three groups during 12 weeks of treatment.

	MET group (*n* = 54)	SPI group (*n* = 53)	COM group (*n* = 51)
Nausea	6 (11.1%)	3 (5.88%)	5 (9.43%)
Diarrhea	3 (1.67%)	2 (3.92%)	3 (5.66%)
Vomiting	1 (1.85%)	0	1 (1.89%)
Polyuria	0	1 (1.96%)	0
Dry mouth	0	2 (3.92%)	0

MET: metformin; SPI: spironolactone; COM: combined.

## Data Availability

The datasets used and/or analyzed during the current study are available from the corresponding author on reasonable request.
